# Seasonal Variations in Soil Enzyme Activity and Nutrient Limitations of Differently Aged *Pinus massoniana* Plantation

**DOI:** 10.3390/microorganisms12112314

**Published:** 2024-11-14

**Authors:** Huiling Chen, Mengmeng Gou, Jianwen Hu, Lei Lei, Sufeng Zhu, Ruyuan Hu, Haiping Zhao, Wenfa Xiao, Changfu Liu

**Affiliations:** 1 Key Laboratory of Forest Ecology and Environment of National Forestry and Grassland Administration, Ecology and Nature Conservation Institute, Chinese Academy of Forestry, No. 2 Dongxiaofu, Xiangshan Road, Haidian District, Beijing 100091, China; 2 Co-Innovation Center for Sustainable Forestry in Southern China, Nanjing Forestry University, Nanjing 210037, China

**Keywords:** *Pinus massoniana*, enzyme activity, nutrient limitation, growing/non-growing season, stand age

## Abstract

Soil extracellular enzymes (SEEs) affect the decomposition of organic matter and microbial nutrient demand. However, the seasonal dynamics of SEE activity for differently aged plantations is still unclear. To analyze the seasonal variations of SEE activity and nutrient limitation for differently aged plantations, this study employed the “space-for-time substitution” method and *Pinus massoniana* plantations of varying ages (6, 13, 29, 38, and 57 years) in subtropical China to determine SEE activity and nutrient limitations in the growing and non-growing seasons. The results showed that SEE activity varied notably with the growth stage and season. In particular, β-1,4-glucosidase activity was higher in the growing season than in the non-growing season, while the opposite was observed for acid phosphatase and leucine-amino-peptidase activity. Acid phosphatase gradually increased with stand age, peaking in the 38-year plantation for the growing and non-growing seasons. Microbial carbon (C)-limitation was higher in the growing season and gradually decreased with forest stand development. Phosphorus (P)-limitation was higher in the growing season than in the non-growing season and was maximum in the 38-year plantation. Moreover, the physicochemical properties and microbial biomass explained the microbial C- and P-limitations, respectively. Compared to the non-growing season, the C- and P-limitations of different stand ages were stronger during the growing season, and the physicochemical properties and microbial biomass were important factors affecting their changes. The study reveals the balance status between soil microorganisms and nutrients in subtropical forest ecosystems and provides guidance for the development of afforestation strategies.

## 1. Introduction

Soil extracellular enzymes (SEEs), as molecular proteins, regulate the transformation and degradation of soil organic matter and affect soil nutrient cycling [1,2]. SEE activity associated with carbon (C), nitrogen (N), and phosphorus (P) cycling can reflect microbial competition for nutrients and their relative resource acquisition strategies [3]. SEE stoichiometry reflects the balances between the availability of nutrients in the environment and the nutrient requirements of the microbial community and between the efficiency of microbial nutrient assimilation and growth [4]. Therefore, SEEs and their stoichiometry are widely used to explore the nutrient status of plantation ecosystems [5,6]. As nutrient availability decreases, microbes produce more SEEs to absorb nutrients from the soil [7]. However, it is still unclear what factors affect the way SEEs receive nutrients from the soil.

SEEs are generally influenced by biotic (e.g., vegetation) and abiotic (e.g., stand age, season, and pH) factors [8,9,10]. Among these, stand age, an indicator of forest structure, directly determines the input of litter, soil microclimate, and phytochemical properties. Furthermore, the interaction of soil, plants, and microorganisms can alter SEE activity and nutrient limitations with stand age [11,12,13]. However, because of the influence of site conditions and tree species, the understanding of nutrient limitations remains unclear [14,15]. Extensive research on SEE activity and nutrient limitation with stand development has found that stand age affects soil nutrient limitation by altering forest material composition and stand structure [14,16]. In terms of nutrient limitations, tree growth is reported to shift from N-limited to P-limited for stand age in subtropical plantations [14]. Despite the importance of this shifting pattern, corresponding studies in forests are limited [17,18].

Season also has an impact on SEE activity and nutrient status [19,20]. The response of SEEs to seasonal changes depends on the impact of changes in the environment (e.g., temperature and humidity) on soil, as well as the applicability of stable C, N, and P resources [21]. The soil environment is a key factor influencing nutrient limitations through microbial activities [4,20]. Previous research reported that an increasing temperature enhances the C- and P-limitations of soil microorganisms in the growing season [22], while a decrease in temperature increases N-limitations [9]. In addition, frequent precipitation increases the limitations of N and P in the soil [23]. Therefore, the main factors affecting nutrient limitations require further investigation. However, to the best of our knowledge, few studies have investigated the effect of the growing and non-growing seasons on the stoichiometric imbalances of soil microbial resources.

*Pinus massoniana*, as one of the main pioneer afforestation tree species in the subtropical regions of China, plays an indispensable part in C sequestration, providing forest resources and facilitating soil and water conservation [24,25]. The total coverage area of *P. massoniana* is reported to be as high as 2.52 × 10^7^ ha [26]. Compared to natural vegetation, the soil quality of planted forests is often lower [27]. Because of the excessive pursuit of rapid and abundant production, *P. massoniana* plantation ecosystems have faced problems such as low stability, reduced productivity, and nutrient limitations [28,29]. However, comprehensive reports on nutrient limitations and the factors affecting nutrient limitation changes in *P. massoniana* plantations across different seasons are lacking. Therefore, this study selected a chronosequence of *P. massoniana* with stand ages of 6, 13, 29, 38, and 57 years to analyze the nutrient limitations and influencing factors. The study aimed to (1) evaluate the effects of stand age and season on SEE activities and nutrient limitations in *P. massoniana* plantations and (2) examine the potential presence of seasonal differences in the influencing factors to determine SEE activities and nutrient limitations.

## 2. Materials and Methods

### 2.1. Study Site and Experiment Design

**Figure 1:**
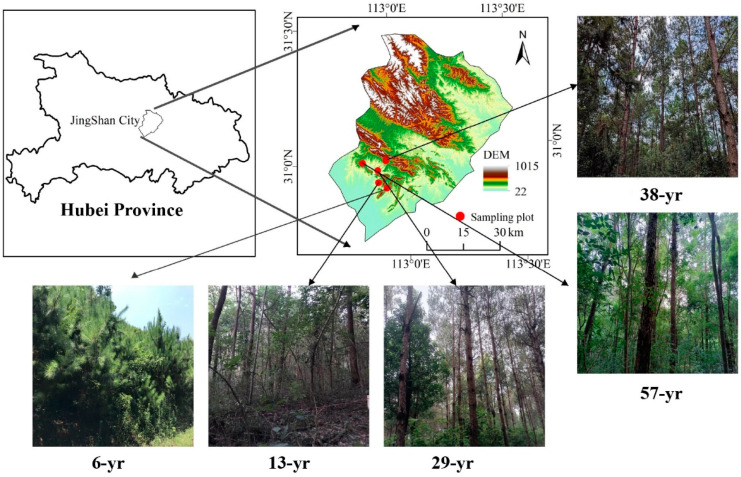
Location of the *Pinus massoniana* plantations study sites, comprising five stand ages.

This study was conducted in Taizishan Forest Farm (112°48′–113°03′ E, 30°48′–31°02′ N) in Hubei province, subtropical China (Figure 1). The climate of the study area is classified as a typical subtropical humid and monsoon climate. Most of the total annual precipitation falls from April to August, with an average annual temperature of 16.4 °C and annual precipitation of 1094.8 mm. Silt-loam yellow soil, classified as the international standard of soil texture classification, which corresponds to Inceptisols in the US soil Taxonomy, is the main soil type in this area [30]. *Cunninghamia lanceolata* and *P. massoniana* are the dominant afforestation tree species in Taizishan. Understory vegetation includes primarily shrubs (i.e., *Sambucus williamsii*, *Ilex chinensis*, and *Vitex negundo var*. *Cannabifolia*) and herbs (i.e., *Crassocephalum crepidioides*, *Smilax china*, and *Rubia cordifolia*) [30].

Based on field investigations, the “space-for-time substitution” method was used to determine *P. massoniana* plantation plots (20 m × 20 m) restored for five age classes (6, 13, 29, 38, and 57 years) [27]. To minimize spatial autocorrelation and edge effects, the distance between the plots was greater than 20 m. Different soil conditions among the plots were considered in the plot determination to improve reliability. Further details of the plot information are described by Shen et al. [31] and Hu et al. [30].

### 2.2. Soil Sampling and Processing

The soil was sampled in the non-growing season (February) and growing season (August) of 2023, with four plots (20 m × 20 m) used as independent replicates for each stand age. Samples were collected with a stainless-steel cylindrical driller from the 0–10 cm layer at 12–15 points in each plot using the “S-sampling” method. The soil samples from each plot were mixed together as composite samples, and visible residues were removed. Each composite sample was passed through a 2 mm mesh and divided into two subsamples. One subsample was stored in a refrigerator at 4 °C to examine SEE activity and microbial biomass and complete the detection within one week. The other subsample was air-dried to determine the physicochemical properties.

### 2.3. Soil Analyses

#### 2.3.1. Measurements of Soil Physicochemical Properties

The soil moisture content (SMC) and pH were measured using oven-drying and electrode methods (soil-to-water ratio of 1:2.5), respectively [31]. The ring knife method was employed to determine the bulk density (BD) and capillary porosity (CP) [32]. Soil organic carbon (SOC) content was quantified by potassium dichromate oxidation–external heating, in which soil total nitrogen (TN) content was measured using the Kjeldahl method [31]. Soil total phosphorus (TP) was determined with a flow injection auto-analyzer (Smartchem450, Inc., AMS, Luoma, Italy) following digestion with H_2_SO_4_-HClO_4_ [32]. Microbial biomass carbon (MBC), microbial biomass nitrogen (MBN), and microbial biomass phosphorus (MBP) were quantified by the chloroform fumigation extraction method [33].

#### 2.3.2. Soil Fluorometric Enzyme Assays

The activities of β-1,4-glucosidase (BG), β-1,4-N-acetylglucosaminidase (NAG), leucine-amino-peptidase (LAP), and acid phosphatase (AP) were measured by fluorimetric enzyme assays, as described by Saiya-Cork and Sinsabaugh et al. [34,35]. SEEs were expressed in units of nmol·h^−1^·g^−1^ dry soil [36]. The enzyme C:N, C:P, and N:P acquisition ratios were estimated using ln(BG):ln(NAG + LAP), ln(BG):ln(AP), and ln(NAG + LAP):ln(AP), respectively [14,29].

Based on the untransformed proportional activities, the vector length and angle of the SEE carriers were predicted to quantify microbial nutritional limitations. The vector length and angle represent the microbial C-limitation degree and N vs. P-limitation, respectively, and are calculated as follows:

(1) $$Vector\;length=SQRT\left(X^{2}+Y^{2}\right)$$

(2) $$Vector\;angle\left({}^{\circ}\right)=Degrees\left(Atan2\left(X,Y\right)\right)$$

where *X* is the relative C vs. P cycling SEE activity and *Y* is the relative C vs. N cycling SEE activity.

Microbial C-limitation increases with the vector length. Vector angles > 45° represent greater P-limitation compared to N-limitation, and vice versa for vector angles < 45°. Microbial P-limitation increases with the vector angle, while the opposite is true for microbial N-limitation [37].

### 2.4. Statistical Analysis

Normality and heterogeneity tests were conducted on all data. Variance and multiple comparisons were then employed to analyze the differences in the influencing factors and nutrient limitations between the growing and non-growing seasons at different stand ages. The relationships between the stand age and nutrient limitations (represented by vector length and vector angle) were examined by linear regression analysis. Spearman’s correlation and the Mantel test were used to investigate the relationship among SEEs, enzyme stoichiometry, soil physicochemical properties, and microbial biomass. Based on the above information, redundancy analysis (RDA) was used to examine vector length, vector angle, and the influence of soil properties. To eliminate collinearity among variables, the Monte Carlo permutation test (999 permutations) and variance inflation factor inspection (VIF < 5) were used to identify valid variables. Partial least squares path modeling (PLS-PM) was conducted using the “plspm” package in R v3.6.3 (https://www.r-project.org/ accessed on 5 November 2024) to determine the effects of the soil factors and nutrient limitations.

## 3. Results

### 3.1. Changes in Soil Physicochemical Properties

The interaction between season and age had significant effects on the ratio of SOC to TP (*p* < 0.05) under different growth periods (Table S1). Under different growth periods, SMC and CP were significantly different in two seasons, and pH, SOC, TN, TP, SOC/TP, and TN/TP were significantly different among different stand ages (*p* < 0.001) (Table S1, Supplementary Materials). SOC was observed to increase and subsequently decrease as the age increased, peaking in the 38-year plantations. TN, TP, pH, and SMC exhibited the opposite trend. The TP content of the 6-year stands was significantly higher than other ages. The lowest pH was observed for the 38-year stands, while that of the other stand ages was higher in the non-growing season (except for 58-year stands). The SMC values in the growing season were significantly higher. This might be attributed to the concentrated local rainfall during the growing season.

### 3.2. Changes in Soil Microbial Biomass and Stoichiometry

The interaction between season and age had significant effects on MBP, MBC/MBN, MBC/MBP, and MBN/MBP (*p* < 0.05) (Table S1). Season and age had significant effects on MBC and MBN (*p* < 0.05) and had extremely significant effects on MBC/MBP and MBN/MBP (*p* < 0.001) (Table S1). MBC and MBP increased and then decreased with stand age, MBC and MBP reached the maximum in the 38-year plantation, and the contents of MBC and MBP in the 13- and 29-year strands were higher in the growing season, while the 38-year and 57-year strands exhibited the opposite trend. In the growing season, MBN increased steadily with age until 29 years, where it reached a maximum and subsequently decreased; in the non-growing season, MBN increased gradually with stand age. (Table 1).

### 3.3. Changes in Soil Enzyme Activity and Stoichiometry

The interaction between season and age had significant effects on NAG (*p* < 0.05) (Table S2). Season had significant effects on BG, LAP, and AP, and age had significant effects on NAG and AP (*p* < 0.05) (Table S2). Significant differences were observed in SEE activity between the non-growing and growing seasons. The BG activity was higher in the growing season, while AP and LAP activities were lower. AP activity gradually increased with the stand age, reaching a peak in the 38-year stands for both seasons. During the growing season, NAG activity in the 6-, 38-, and 57-year stands exceeded that in the non-growing season, and the difference was significant for the 57-year stands. The ratios lnBG:ln(NAG + LAP) and lnBG:lnAP were higher in the growing season, while the ln(NAG + LAP):lnAP ratio was higher in the non-growing season, and the ratio of the 38-year stands was significantly lower than other ages (Table 2).

**Figure 2:**
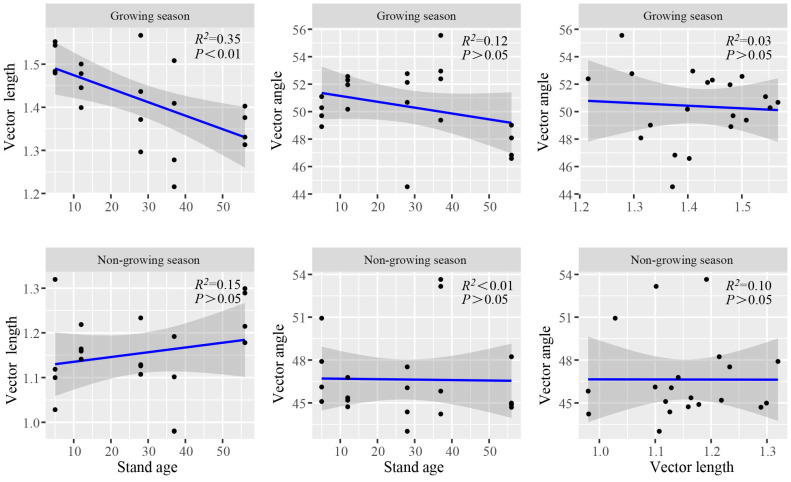
Linear regression analysis used to identify the relationship between soil microbial C-limitation (vector length), microbial P-limitation (vector angle), and stand age in the growing and non-growing seasons and the relationship between microbial C-limitation and N-limitation in the growing and non-growing seasons among five stand ages.

Vector analysis revealed that season had a significant impact on nutrient limitations (Table S2). The vector length of each age was significantly higher in the growing season compared to the non-growing season. This indicates that the microbial C-limitation in the former was significantly stronger than in the latter. The vector length decreased significantly with stand age in the growing season, revealing that the microbial C-limitation weakened gradually with age. During the non-growing season, C-limitation exhibited the following trend: 57-year > 13-year > 29-year > 6-year > 38-year. In terms of the vector angle, the P-limitation was significantly stronger in the growing season observations. P-limitation exhibited the following trend during the growing season: 38-year > 13-year > 29-year > 6-year > 57-year; and 38-year > 6-year > 57-year > 13-year > 29-year in the non-growing season. P-limitation was strongest for the 38-year strands for both seasons (Table 2). Based on the linear regression analysis, microbial C-limitation and N/P-limitation did not exhibit any clear relationships in the growing and non-growing seasons (Figure 2).

### 3.4. Relationships Between Soil Physicochemical Properties, Microbial Biomass, and Enzyme Activity

**Figure 3:**
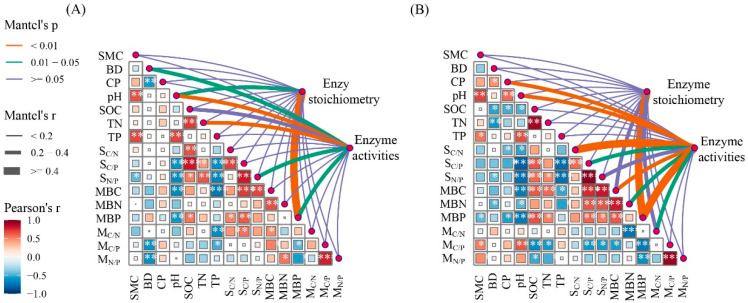
Spearman’s correlations of soil enzyme activities, extracellular enzyme stoichiometry, soil properties, and microbial biomass. The thicknesses of the lines indicate the strength of the correlation. (**A**) Growing; (**B**) non-growing. SMC, BD, CP, pH, SOC, TN, TP, MBC, MBN, and MBP represent the abbreviations of soil moisture content, bulk density, capillary porosity, pH, soil organic carbon, total nitrogen, total phosphorus, microbial biomass carbon, microbial biomass nitrogen, and microbial biomass phosphorus, respectively. S_C/N_: SOC:TN; S_C/P_: SOC:TP; S_N/P_: TN:TP; M_C/N_: MBC: MBN; M_C/P_: MBC: MBP; M_N/P_: MBN: MBP. Enzyme stoichiometry: E_C/N_: lnBG:ln(NAG + LAP); E_C/P_: lnBG:lnAP; E_N/P_: ln(NAG + LAP):lnAP. Enzymatic activities: BG: β-1,4-glucosidase; NAG: β-1,4-N-acetylglucosaminidase; LAP: leucine-amino-peptidase; AP: acid phosphatase. * *p* < 0.05, ** *p* < 0.01, *** *p* < 0.001.

**Figure 4:**
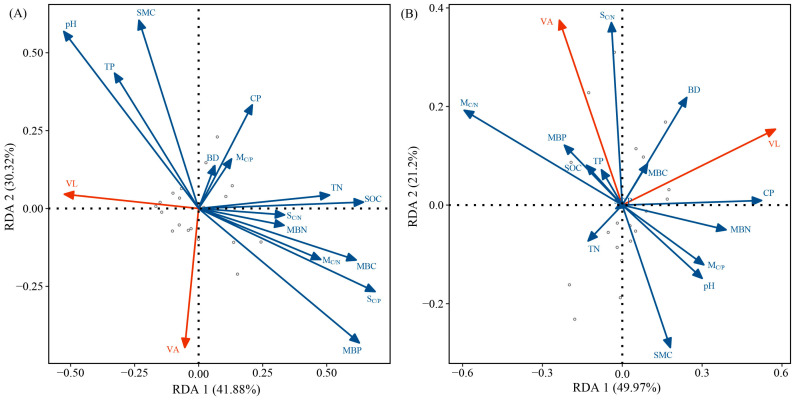
Redundancy analysis (RDA) was used to identify the relationships among the vector length, vector angle (VL, VA) (red arrow), physicochemical properties, and microbial biomass (blue arrow). (**A**) Growing season; (**B**) non-growing season.

The Mantel tests indicate that pH and MBP were key factors affecting enzyme activity and enzyme stoichiometry in the growing season; MBP was a key factor affecting enzyme activity and enzyme stoichiometry in the non-growing season (Figure 3). BD, TN, and TN/TP had a certain impact on enzyme activity in the growing season. CP, pH, SOC/TN, SOC/TP, and microbial biomass had a certain influence on enzyme activity in the non-growing season (Figure 3). The RDA results show that during the growing season, the first axis explains 41.88% of the variables related to vector length and angle, while the second axis explains 30.32% of the variables (Figure 4A). During the non-growing season, the first axis explains 49.97% of the variables related to vector length and angle, while the second axis explains 21.2% of the variables (Figure 4B).

### 3.5. Driving Factors Affecting Soil Microbial Metabolic Limitation

**Figure 5:**
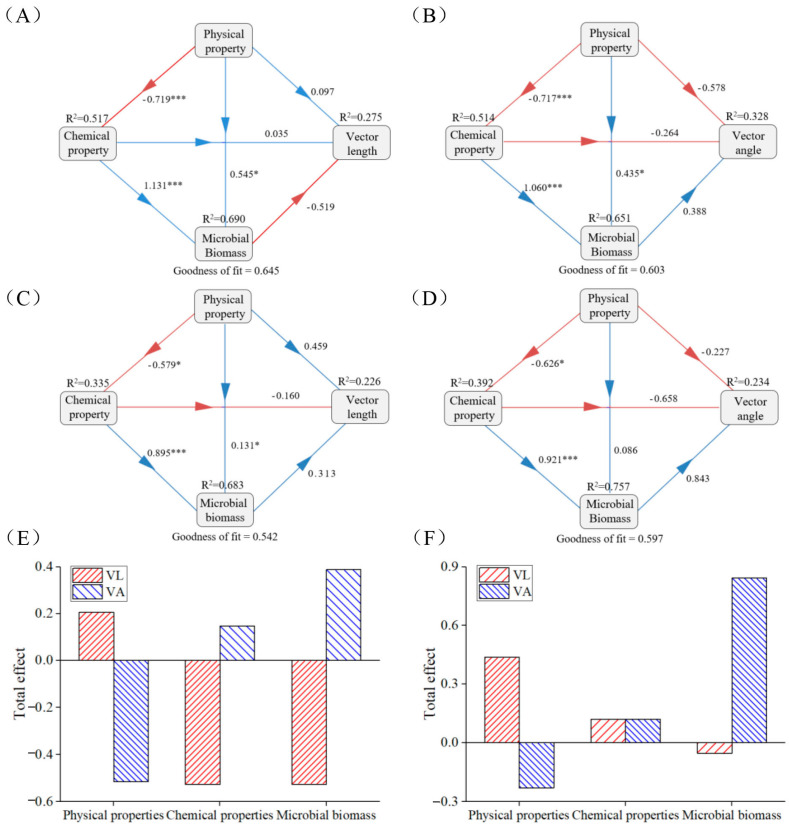
The partial least squares path model (PLS-PM) showing the direct, indirect, and total effect of growing (**A**,**B**,**E**) and non-growing (**C**,**D**,**F**) soil physical property, chemical property, and microbial biomass on the microbial C-limitation (vector length) and microbial N-limitation (vector angle). The red and blue arrows indicate the positive and negative flows of causality, respectively (*p* < 0.05). The number on the arrow indicates the effective normalized path coefficient. Physical properties include soil moisture content, bulk density, and capillary porosity. Chemical properties include pH, soil organic carbon, total nitrogen, and total phosphorus. Microbial biomass includes soil microbial biomass carbon, microbial biomass nitrogen, and microbial biomass phosphorus. * *p* < 0.05, *** *p* < 0.001.

PLS-PM analysis indicated that the effects of biotic and abiotic factors on nutrient limitations were consistent among the seasons. The physicochemical properties had a positive effect on microbial C-limitation, while microbial biomass had negative effects. The opposite was observed for the effects of physicochemical properties and microbial biomass on microbial P-limitation. The chemical properties (during the growing season) and physical properties (during the non-growing season) exerted the greatest total effect on microbial C-limitation, and microbial biomass had the strongest negative total effect on microbial P-limitation (0.388 and 0.843 for the growing and non-growing seasons, respectively). Therefore, physicochemical properties and microbial biomass were the major factors affecting microbial C-limitation and P-limitation, respectively (Figure 5).

## 4. Discussion

### 4.1. Response of Soil Physicochemical Properties to Stand Age and Season

As an important index of forest structure [12], stand age plays a crucial driving role in canopy density and understory vegetation composition, altering the penetration of solar radiation, as well as the type, quantity, and quality of stand litter, thus changing SOC dynamics [38,39,40]. In this study, SOC increased with stand age. This is attributed to the reduction in stand density with increasing stand age. At a suitable stand density level, the competitive pressure between stands decreases [41], and soil water and temperature facilitate microbial activity [32,42,43]. This accelerates the material cycle, allowing soils to store additional carbon [32,44]. The increase in stand age also reflects an increase in C input via photosynthesis and the accelerated degradation of litter, with a direct impact on SOC levels [38,40]. Because of the rapid growth of *P. massoniana*, it absorbed more soil phosphorus for growth, resulting in the phosphorus content in the soil of *P. massoniana* plantations being relatively lower in the growing season [45,46]. According to the relative absorption hypothesis, when *P. massoniana* leaves fall during the non-growing season, plants return a large amount of phosphorus to the soil, increasing soil phosphorus content [47]. MBC is a key index for the soil microclimate (e.g., soil moisture, pH, soil nutrient status, and vegetation type) [13,39]. In this study, the MBC content in 5-year stands was significantly lower than in other ages (Table 1). This may be attributed to the lower vegetation diversity of understory shrub and herb layers in the 5-year stands, resulting in a lower MBC content [39,48].

### 4.2. Response of Soil Enzyme Activity and Nutrient Limitations to Stand Age and Season Development

Age and season influence microbial SEE activity; enzyme C, N, and P acquisition rates; and nutrient limitations. The quality of litter increases with stand age; it accelerates the decomposition of litter through the effect of microbial, leading to more nutrients returning to the soil and changing the soil nutrient limitations [49], while seasonal adjustment is usually followed by changes in temperature, humidity, etc. [50,51]. The nutrient requirements and utilization strategies of soil microorganisms, as well as SEE activities, adjust in response to the complex series of environmental changes induced by temperature and humidity [9,32,52,53]. Furthermore, soil microbial communities secrete various SEEs to catalyze the decomposition of soil organic matter. During forest development, the composition, structure, and function of soil microbial communities change, and these changes will affect the activity and types of SEEs, resulting in changes in soil nutrient limitations [54]. We observed age and season to significantly regulate the acquisition rates of enzymes C, N, and P. At the same growth stage, the activity of LAP and AP were higher in the 13, 29, and 37-year (Table 2). This indicates an enhancement in the N and P acquisition capacity of soil microorganisms [55,56]. Season has a strong impact on the acquisition rates of enzymes C, N, and P. In the growing season, the temperature is higher, and the acquisition rates of C and P by soil microorganisms are significantly higher than those in the non-growing season (Table 2). Therefore, microorganisms are more inclined to acquire C and N in the growing season [9]. A large number of nutrients are required to maintain normal microorganism activity, and they metabolize rapidly to absorb carbon and nitrogen from the soil to meet their needs [32,53,57,58]. In summary, soil SEE activity varies across different growth cycles and seasons, highlighting the impact of stand age and season on SEE synthesis. Moreover, the nutrient preferences of the microbial communities also influence the changes in soil SEEs, thereby altering C- and N-limitations [59]. According to the research results, the C-limitation in the growing season was greater than in the non-growing season (Table 2, Figure 2). Therefore, management should be strengthened to alleviate C-limitation in the growing season.

### 4.3. Soil Enzyme Activity and the Nutrient Dynamic Balance with Stand Age and Season Development

In the process of stand development and growth cycle replacement, when the nutrient supply is unbalanced, the secretion of SEE is regulated by microorganisms to meet the nutrient demand of the stand [60,61,62], and microorganisms have an advantage over plants in terms of nutrient acquisition [63]. Plants change nutrient availability by regulating microbial enzyme production [60,64], while microorganisms not only rely on SEEs produced by plant roots but also obtain the energy needed for production and growth through degradation products of litter and soil microbial secretions [36].

Soil pH value is an important factor affecting SEEs and nutrient limitation and varies with the type of SEE (Figure 3, Figure 4 and Figure 5) [65,66]. The pH of tropical and subtropical forest soil is mainly acidic, with a range of 5.1–6.3 (Table 1). Low pH indicates the presence of highly weathered conditions, which reduces the migration of some anions in the soil. In low pH and low phosphorus soil, soil microorganisms secrete more AP to meet the demand for phosphorus, and thus, AP exhibits a significant negative correlation with pH [67,68]. The influence of pH on SEE activity also reflects the climate-induced changes in soil properties, microorganisms, etc. Our results indicate that SMC changed significantly with stand age (Table 1). In the growing season, appropriate SMC and temperature increase the nutrient requirements of microorganisms and indirectly affect nutrient limitations (Table 1) [60]. The RDA and PLS-PM results indicate that TP and MBC were the main influencing factors affecting SEE activity and nutrient limitation (Figure 4 and Figure 5), which is consistent with the results of previous studies [13,29,69]. Because the *P. massoniana* needs to absorb more phosphorus for its own growth, which leads to changes in the phosphorus content in the soil [45,46], the change in vegetation diversity in the understory shrub and herb layers leads to the change in MBC [39]. Changes in TP and MBC have a certain effect on the nutrient limitation of *P. massoniana* plantation. With the colonization and growth of plants and microorganisms, the supply and demand balance of the C cycle in plantations changes, exhibiting a short supply [70]. The continuous input of the plant carbon pool improves soil carbon storage conditions and protects the reproduction of microorganisms in the soil [71]. In this study, with stand age, soil microbial C-limitation gradually increased in the non-growing season and gradually decreased in the growing season (Figure 2). This indicates that the relative energy required by microorganisms gradually increased in the non-growing season and decreased in the growing season. The input of residual roots and leaves increases in the non-growing season, resulting in relatively abundant soil carbon sources. However, the soil loss in the study area was severe, accelerating the loss of carbon [72]. In addition, microbial C-limitation in *P. massoniana* was significantly higher in the growing season, revealing that temperature and other factors affect the utilization efficiency of soil microorganisms on C [9]. Microbial P-limitations were observed at all stand ages across both seasons, which is the same as the results of Jian and Xu [29,73], indicating the presence of P-limitation in tropical and subtropical regions.

## 5. Conclusions

Soil extracellular enzyme stoichiometry was used to explore the nutrient limitations in *P. massoniana* plantations of different ages and seasons, which increased our understanding of soil nutrient management of *P. massoniana* plantations. The microbial metabolism of different ages *P. massoniana* plantations was limited by C and P. In terms of plant-microbial nutrient competition, the C- and P-limitations of microorganisms were stronger in the growing season and decreased with stand age. Soil physicochemical properties (pH, TP, and CP) and microbial biomass (MBC and MBP) are the main driving factors in determining nutrient limitations. In conclusion, there are common C- and P-limitations in *P. massoniana* plantations, while there is a higher C-limitation in the growing season. Therefore, it is necessary to strengthen management (e.g., fertilization, pruning, etc.) to enhance productivity in the growing season.

## Figures and Tables

**Table 1:** Physical and chemical properties in the growing and non-growing season soils of *Pinus massoniana* plantations with five stand ages.

Parameter	6-Year		13-Year		29-Year		38-Year		57-Year	
	Growing	Non-Growing	Growing	Non-Growing	Growing	Non-Growing	Growing	Non-Growing	Growing	Non-Growing
SMC(%)	25.83 ± 2.58 Aa	22.18 ± 0.98 Aa	22.62 ± 0.49 Aa	20.78 ± 1.17 Aa	21.52 ± 1.22 Aa	15.45 ± 0.41 Bb	21.75 ± 0.91 Aa	17.52 ± 1.51 Aab	26.25 ± 2.00 Aa	20.75 ± 2.41 Aa
BD(g/cm^3^)	1.37 ± 0.01 Aa	1.38 ± 0.02 Aa	1.21 ± 0.03 Ba	1.31 ± 0.02 Aa	1.35 ± 0.05 Aa	1.39 ± 0.04 Aa	1.38 ± 0.08 Aa	1.28 ± 0.05 Aa	1.29 ± 0.07 Aa	1.34 ± 0.06 Aa
CP(%)	40.76 ± 0.26 Ba	56.03 ± 1.23 Aa	41.24 ± 0.87 Ba	54.23 ± 1.53 Aab	41.89 ± 0.69 Ba	53.89 ± 2.61 Aab	40.36 ± 0.92 Ba	49.41 ± 1.58 Ab	43.67 ± 2.76 Ba	56.46 ± 0.68 Aa
pH	6.28 ± 0.04 Ba	6.66 ± 0.07 Aa	5.66 ± 0.08 Ab	5.83 ± 0.05 Ab	5.11 ± 0.17 Ac	5.30 ± 0.22 Acd	4.90 ± 0.07 Ac	4.95 ± 0.05 Ad	5.92 ± 0.16 Ab	5.73 ± 0.26 Abc
SOC(g/Kg)	15.31 ± 0.53 Ab	16.88 ± 0.66 Ac	14.63 ± 0.17 Bb	16.50 ± 0.68 Ac	15.47 ± 0.34 Ab	18.58 ± 2.22 Abc	24.11 ± 1.22 Aa	22.90 ± 0.56 Aa	23.87 ± 2.09 Aa	21.74 ± 1.21 Aab
TN(g/Kg)	1.52 ± 0.10 Aab	1.49 ± 0.06 Abc	1.30 ± 0.09 Ab	1.33 ± 0.07 Ac	1.35 ± 0.17 Ab	1.48 ± 0.14 Abc	1.76 ± 0.15 Aa	1.82 ± 0.12 Aa	1.76 ± 0.05 Aa	1.78 ± 0.08 Aab
TP(g/Kg)	0.34 ± 0.02 Aa	0.38 ± 0.03 Aa	0.21 ± 0.01 Ab	0.22 ± 0.01 Ab	0.21 ± 0.02 Ab	0.22 ± 0.01 Ab	0.22 ± 0.01 Ab	0.26 ± 0.01 Ab	0.24 ± 0.02 Ab	0.25 ± 0.02 Ab
S_C/N_	10.15 ± 0.45 Ab	11.37 ± 0.25 Ab	11.42 ± 0.7 Aab	12.46 ± 0.13 Aab	11.91 ± 1.25 Aab	12.48 ± 0.42 Aab	13.88 ± 0.58 Aa	12.73 ± 0.70 Aa	13.56 ± 1.01 Aa	12.17 ± 0.30 Aab
S_C/P_	45.84 ± 4.01 Ac	44.69 ± 3.51 Ab	71.19 ± 2.56 Ab	75.57 ± 4.23 Aa	75.78 ± 6.79 Ab	83.00 ± 8.13 Aa	107.31 ± 2.47 Aa	89.11 ± 2.35 Ba	98.10 ± 5.37 Aa	85.74 ± 1.69 Aa
S_N/P_	4.58 ± 0.59 Ab	3.93 ± 0.28 Ab	6.30 ± 0.40 Aab	6.08 ± 0.39 Aa	6.56 ± 0.88 Aa	6.61 ± 0.42 Aa	7.79 ± 0.45 Aa	7.07 ± 0.48 Aa	7.30 ± 0.43 Aa	7.06 ± 0.23 Aa
MBC(mg/Kg)	28.39 ± 1.88 Bb	132.13 ± 8.49 Ac	254.61 ± 26.83 Aa	260.66 ± 16.33 Ab	299.06 ± 46.11 Aa	268.65 ± 16.66 Ab	279.87 ± 21.1 Aa	366.22 ± 42.45 Aa	277.40 ± 33.49 Aa	344.79 ± 2.07 Aa
MBN(mg/Kg)	15.32 ± 7.14 Ab	17.02 ± 5.04 Ab	26.21 ± 3.85 Bab	57.12 ± 4.06 Aa	37.95 ± 9.96 Aa	49.51 ± 5.59 Aa	25.46 ± 3.82 Bab	51.38 ± 6.32 Aa	26.47 ± 3.74 Bab	55.56 ± 7.95 Aa
MBP(mg/Kg)	1.05 ± 0.27 Ab	0.22 ± 0.02 Bc	0.71 ± 0.08 Ab	0.35 ± 0.03 Bc	1.47 ± 0.29 Ab	2.17 ± 0.18 Ab	4.89 ± 0.84 Aa	6.67 ± 0.81 Aa	1.62 ± 0.21 Ab	2.16 ± 0.20 Ab
M_C/N_	4.57 ± 2.20 Ab	9.42 ± 2.17 Aa	10.76 ± 2.62 Aa	4.57 ± 0.04 Ab	8.81 ± 1.29 Aab	5.55 ± 0.40 Ab	11.42 ± 0.98 Aa	7.15 ± 0.08 Bab	10.62 ± 0.48 Aa	6.71 ± 1.20 Bab
M_C/P_	30.77 ± 5.06 Bd	616.62 ± 67.22 Aa	378.64 ± 73.60 Ba	772.51 ± 103.52 Aa	212.27 ± 25.66 Ab	127.84 ± 17.56 Bb	65.40 ± 15.75 Acd	57.24 ± 9.11 Ab	181.76 ± 36.08 Abc	163.81 ± 13.83 Ab
M_N/P_	13.47 ± 6.10 Bbc	77.03 ± 22.12 Ab	38.13 ± 7.28 Ba	169.42 ± 23.51 Aa	25.76 ± 5.05 Aab	23.54 ± 3.86 Ac	6.25 ± 2.07 Ac	8.05 ± 1.36 Ac	17.58 ± 4.30 Abc	26.09 ± 3.94 Ac

SMC: soil moisture content; BD: bulk density; CP: capillary porosity; SOC: soil organic carbon; TN: total nitrogen; TP: total phosphorus; S_C/N_: SOC:TN; S_C/P_: SOC:TP; S_N/P_: TN:TP; MBC: microbial biomass carbon; MBN: microbial biomass nitrogen; MBP: microbial biomass phosphorus; M_C/N_: MBC: MBN; M_C/P_: MBC: MBP; M_N/P_: MBN: MBP. Significant differences among different season levels of the same age are identified with A and B (*p* < 0.05); significant differences among different age levels of the same season are identified with a, b, c, and d (*p* < 0.05). Values are the mean with standard error (SE), where *n* = 4.

**Table 2:** Extracellular enzyme activities and extracellular enzyme stoichiometry in the growing and non-growing season soils of *Pinus massoniana* plantations with five stand ages.

Parameter		6-Year		13-Year		29-Year		38-Year		57-Year	
		Growing	Non-Growing	Growing	Non-Growing	Growing	Non-Growing	Growing	Non-Growing	Growing	Non-Growing
EEA	BG (nmol·h^−1^ g^−1^ dry soil)	158.67 ± 5.11 Aa	79.99 ± 20.11 Bbc	127.15 ± 8.08 Aa	126.04 ± 9.15 Aab	133.62 ± 23.26 Aa	110.08 ± 4.67 Aabc	126.72 ± 40.04 Aa	67.89 ± 3.48 Ac	137.05 ± 26.01 Aa	155.38 ± 25.24 Aa
	NAG (nmol·h^−1^ g^−1^ dry soil)	64.48 ± 3.52 Ab	48.51 ± 8.05 Ab	62.91 ± 1.70 Bb	98.41 ± 5.20 Aa	74.39 ± 15.36 Ab	115.63 ± 8.89 Aa	70.62 ± 16.39 Ab	58.48 ± 11.04 Ab	121.9 ± 25.43 Aa	94.15 ± 11.54 Aa
	LAP (nmol·h^−1^ g^−1^ dry soil)	14.76 ± 4.74 Ba	126.11 ± 33.05 Aa	6.63 ± 3.04 Ba	228.39 ± 15.21 Aa	17.36 ± 2.96 Ba	230.36 ± 54.92 Aa	18.32 ± 8.66 Aa	204.01 ± 100.69 Aa	13.98 ± 3.44 Ba	193.31 ± 29.84 Aa
	AP (nmol·h^−1^ g^−1^ dry soil)	182.39 ± 6.96 Ba	260.00 ± 6.43 Ac	216.07 ± 10.09 Ba	361.11 ± 11.10 Ab	304.10 ± 137.08 Aa	345.04 ± 43.22 Ab	394.45 ± 113.51 Aa	460.84 ± 30.29 Aa	212.57 ± 29.78 Ba	315.04 ± 4.28 Abc
EES	E_C/N_	1.16 ± 0.02 Aa	0.84 ± 0.05 Ba	1.14 ± 0.02 Aa	0.84 ± 0.01 Ba	1.09 ± 0.06 Aab	0.82 ± 0.03 Ba	1.07 ± 0.04 Aab	0.81 ± 0.07 Ba	1.00 ± 0.01 Ab	0.89 ± 0.02 Ba
	E_C/P_	0.97 ± 0.01 Aa	0.77 ± 0.05 Bb	0.90 ± 0.01 Aab	0.82 ± 0.02 Bab	0.91 ± 0.05 Aab	0.81 ± 0.01 Aab	0.82 ± 0.06 Ab	0.69 ± 0.01 Ac	0.91 ± 0.02 Aab	0.87 ± 0.03 Aa
	E_N/P_	0.84 ± 0.01 Aab	0.92 ± 0.04 Aa	0.79 ± 0.02 Bb	0.98 ± 0.02 Aa	0.84 ± 0.06 Aab	0.99 ± 0.03 Aa	0.77 ± 0.04 Ab	0.87 ± 0.08 Aa	0.91 ± 0.02 Aa	0.98 ± 0.03 Aa
Vector	length	1.51 ± 0.02 Aa	1.14 ± 0.06 Bab	1.46 ± 0.02 Aab	1.17 ± 0.02 Bab	1.42 ± 0.06 Aab	1.15 ± 0.03 Bab	1.35 ± 0.07 Ab	1.06 ± 0.05 Bb	1.36 ± 0.02 Ab	1.25 ± 0.03 Ba
	angle	50.00 ± 0.46 Aab	47.51 ± 1.28 Aa	51.75 ± 0.54 Aa	45.52 ± 0.44 Ba	50.02 ± 1.88 Aab	45.24 ± 0.98 Aa	52.57 ± 1.27 Aa	49.22 ± 2.44 Aa	47.63 ± 0.57 Ab	45.70 ± 0.85 Aa

EEA: extracellular enzyme activities; EES: extracellular enzyme stoichiometry; BG: β-1,4-glucosidase; NAG: β-1,4-N-acetylglucosaminidase; LAP: leucine aminopeptidase; AP: acid phosphatase; E_C/N_: Ln(BG):Ln(NAG + LAP); E_C/P_: Ln(BG):Ln(AP); E_N/P_: Ln(NAG + LAP):Ln(AP). Significant differences among different season levels of the same age are identified with A and B (*p* < 0.05); significant differences among different age levels of the same season are identified with a, b, and c (*p* < 0.05). Values are the mean with standard error (SE), where *n* = 4.

## Data Availability

The original contributions presented in the study are included in the article/Supplementary Materials, further inquiries can be directed to the corresponding authors.

## Supplementary Materials

The following supporting information can be downloaded at: https://www.mdpi.com/article/10.3390/microorganisms12112314/s1, Table S1: The significance of soil physicochemical properties in the growing and non-growing season of *P. massoniana* plantations with different ages; Table S2: The significance of extracellular enzyme activities and extracellular enzyme stoichiometry in the growing and non-growing season soil of *P. massoniana* plantations with different ages.
