# Supplementary material for: Seasonal Variations in Soil Enzyme Activity and Nutrient Limitations of Differently Aged Pinus massoniana Plantation
Source: Microorganisms. 2024 Nov 14;12(11):2314. doi: 10.3390/microorganisms12112314 (PMC11596497; doi:10.3390/microorganisms12112314)
Supplement: Supplementary file 1 [file microorganisms-12-02314-s001.zip › microorganisms-3288602-supplementary.pdf]

Supplementary materials

Table S1

The significance of soil physicochemical properties in the growing and non-growing season of *P. massoniana* plantations with different ages.

Parameter	Factor	Significance	Parameter	Factor	Significance
SMC (%)	S	***	$S_{C/P}$	S	ns
	Y	**		Y	***
	S*Y	ns		S*Y	**
BD (g/cm^3)	S	ns	$S_{N/P}$	S	ns
	Y	ns		Y	***
	S*Y	ns		S*Y	ns
CP (%)	S	***	MBC (mg/Kg)	S	**
	Y	**		Y	***
	S*Y	ns		S*Y	ns
pH	S	ns	MBN (mg/Kg)	S	***
	Y	***		Y	***
	S*Y	ns		S*Y	ns
SOC (g/Kg)	S	ns	MBP (mg/Kg)	S	ns
	Y	***		Y	***
	S*Y	ns		S*Y	**
TN (g/Kg)	S	ns	$M_{C/N}$	S	**
	Y	***		Y	ns
	S*Y	ns		S*Y	**
TP (g/Kg)	S	**	$M_{C/P}$	S	***
	Y	***		Y	***
	S*Y	ns		S*Y	***
$S_{C/N}$	S	ns	$M_{N/P}$	S	***
	Y	**		Y	***
	S*Y	ns		S*Y	***

Note: SMC: soil moisture content; BD: bulk density; CP: capillary porosity; SOC: soil organic carbon; TN: total nitrogen; TP: total phosphorus; $S_{C/N}$: SOC:TN; $S_{C/P}$: SOC:TP; $S_{N/P}$: TN:TP; MBC: microbial biomass carbon; MBN: microbial biomass nitrogen; MBP: microbial biomass phosphorus; $M_{C/N}$: MBC: MBN; $M_{C/P}$: MBC: MBP; $M_{N/P}$: MBN: MBP; S: Season; Y: Year; ** represent $p \leq 0.05$, *** represent $p \leq 0.001$, ns represent $p > 0.05$.

Table S2

The significance of extracellular enzyme activities and extracellular enzyme stoichiometry in the growing and non-growing season soil of *P. massoniana* plantations with different ages.

Parameter	Factor	Significance	Parameter	Factor	Significance
BG	S	**		S	***
(nmol·h^{-1}g^{-1}	Y	ns	$E_{C/P}$	Y	**
dry soil)	S*Y	ns		S*Y	ns
NAG	S	ns		S	***
(nmol·h^{-1}g^{-1}	Y	**	$E_{N/P}$	Y	**
dry soil)	S*Y	**		S*Y	ns
LAP	S	***		S	***
(nmol·h^{-1}g^{-1}	Y	ns	Vector length	Y	ns
dry soil)	S*Y	ns		S*Y	ns
AP	S	**		S	***
(nmol·h^{-1}g^{-1}	Y	**	Vector angle	Y	**
dry soil)	S*Y	ns		S*Y	ns
	S	***	/	/	/
$E_{C/N}$	Y	ns	/	/	/
	S*Y	ns	/	/	/

Note: BG: β-1,4-glucosidase; NAG: β-1,4-N-acetylglucosaminidase; LAP: leucine aminopeptidase; AP: acid phosphatase; $E_{C/N}$: Ln(BG):Ln(NAG + LAP); $E_{C/P}$: Ln(BG):Ln(AP); $E_{N/P}$: Ln(NAG + LAP):Ln(AP). S: Season; Y: Year; ** represent $p \leq 0.05$, *** represent $p \leq 0.001$, ns represent $p > 0.05$.
